# Lobular intraepithelial neoplasia arising within breast fibroadenoma

**DOI:** 10.1186/1756-0500-6-267

**Published:** 2013-07-12

**Authors:** Gennaro Limite, Emanuela Esposito, Viviana Sollazzo, Giuseppe Ciancia, Rosa Di Micco, Dario De Rosa, Pietro Forestieri

**Affiliations:** 1 University Department of Clinical Medicine and Surgery, Breast Unit, University of Naples Federico II, Naples, Italy; 2 Department of Pathology, University of Naples Federico II, Naples, Italy; 3 Department of Diagnostic Radiology, University of Naples Federico II, Naples, Italy

**Keywords:** Fibroadenoma, Lobular intraepithelial neoplasia, Popcorn-like calcifications

## Abstract

**Background:**

Fibroadenomas are the second most common breast pathology occurring in young women under the age of 35 years old. Fibroadenomas can be classified as simple or complex according to histological features. Complex fibroadenomas differ from simple fibroadenomas because of the presence of cysts (3 mm), sclerosing adenosis, epithelial calcifications, or papillary apocrine changes. Most fibroadenomas are clinically identifiable. In 25% of cases, fibroadenomas are non-palpable and are diagnosed with mammography and ultrasound. Differential diagnosis with well differentiated breast cancer is often necessary, particularly with medullary or mucinous tumors. Calcification findings within fibroadenomas by mammogram have to be investigated. The age of a lump is usually reflected by calcifications. Microcalcification can hide foci of carcinoma in situ when they are small, branching type, and heterogeneous. However, many morphological possibilities may not be reliable for deciding whether a certain calcification is the product of a malignant or a benign process. From a radiological point of view, fibroadenomas containing foci of carcinoma in situ can be indistinguishable from benign lesions, even if the incidence of carcinoma within fibroadenomas is estimated as 0.1–0.3%, and it could be a long-term risk factor for invasive breast cancer.

**Case presentation:**

A 44-year-old woman presented with a 1.5-cm palpable, smooth, mobile lump in the lower-inner quadrant of her right breast. Standard mediolateral oblique and craniocaudal mammograms showed a cluster of eccentric popcorn-like calcifications within the fibroadenoma. After lumpectomy, a definitive histological examination confirmed the intra-operative diagnosis of a benign mass. However, lobular intraepithelial neoplasia foci were found, surrounded by atypical lobular hyperplasia.

**Conclusions:**

The possibility of an old benign breast lump might be supported by fine needle aspiration biopsy or core biopsy before initiating follow-up. According to our experience, when patients are older than 40 years and have a familial history of breast cancer, we prefer to carry out lumpectomy with follow up to avoid the risk of underestimation in situ foci within the lump.

## Background

Fibroadenomas are the second most common breast pathology, following fibrocystic disease, and occur in women younger than 35 years old. The peak age of incidence is in the third decade. Fibroadenoma is a benign breast tumor that is effectively treated by local excision consisting of lumpectomy. Fibroadenomas can be classified as simple or complex according to histological features. Complex fibroadenomas differ from simple fibroadenomas because of the presence of cysts (>3 mm), sclerosing adenosis, epithelial calcifications, or papillary apocrine changes. The stroma in young fibroadenomas is usually mostly cellular, and as these lumps age they tend to become less cellular and more sclerotic. Epithelial elements atrophy and calcification often develops. Most fibroadenomas are clinically identifiable, whereas in 25% of cases, fibroadenomas are non-palpable and are diagnosed with mammography and ultrasound [1]. Differential diagnosis with well differentiated breast cancer is often necessary, particularly with medullary or mucinous tumors. Calcification findings within fibroadenomas by mammogram often need to be investigated. The age of a lump is usually reflected by calcifications. Calcifications of the breast are the smallest structure identified on a mammogram and are always a sign of a previous or ongoing breast tissue alteration. Calcifications are usually benign and their frequency increases with age. Active cell secretion, cellular necrotic debris, inflammation, trauma or radiation are the most usual reasons for the formation of calcifications. To standardize mammographic reporting, the American College of Radiology implemented the Breast Imaging Reporting and Database System (BIRADS). For calcifications, BIRADS takes into account distribution within the breast and patterns. Calcifications may be a sign of benign changes but they can also disclose a yet nonpalpable malignant process. There are “benign-looking” calcifications that may show a malignant process and “suspicious-looking” findings that may be less malignant than “benign-looking” ones. Regardless of the final diagnosis, calcifications should be assessed according to shape, size, density, number, distribution, and associated findings. Morphology alone in some well-defined instances (popcorn, eggshell or tram-track calcification) can be used to identify the benign nature of a breast process. However, certain morphologies (branching and casting types) will always be highly suspicious. However, there are many morphological possibilities, which are unreliable for deciding whether a certain calcification is the product of a malignant or a benign process [2]. Accordingly, popcorn-like large (>2 mm) calcifications may be considered “benign-looking”, whereas small, branching type and heterogeneous calcifications can express foci of carcinoma in situ. From a radiological point of view, fibroadenoma containing foci of carcinoma in situ can be indistinguishable from benign lesions, even if the incidence of carcinoma within fibroadenomas is estimated as 0.1–0.3% [3]. Moreover, it could be a long-term risk factor for invasive breast cancer. Seven studies have attempted to confirm the breast cancer risk associated with fibroadenomas. Dupont et al. and McDivitt et al. are considered to provide the strongest evidence, and showed that the relative risk for excised fibroadenomas without hyperplasia ranges from 1.48–1.7, that for fibroadenomas with hyperplasia is 3.47–3.7, and that for fibroadenomas with hyperplasia and atypia is 6.9–7.29, persisting for more than 20 years [4,5]. The other five studies by Levi et al., Ciatto et al., Moskowitz et al., Carter et al., and Levshin et al., are considered to provide weaker evidence, although they showed similar results [6,7]. None of these results could be used to quantify the risk of excised, non-excised, and asymptomatic fibroadenoma. The overall risk of invasive breast cancer is 2.17 times higher among patients with complex fibroadenomas than among controls (95% confidence interval [CI], 1.5 to 3.2) [4]. The relative risk increases to 3.10 among patients with complex fibroadenomas (95% CI, 1.9 to 5.1) and remains elevated for decades after diagnosis. Patients with benign proliferative disease in the parenchyma adjacent to the fibroadenoma have a relative risk of 3.88 (95% CI, 2.1 to 7.3). Patients with a familial history of breast cancer in whom complex fibroadenomas are diagnosed have a relative risk of 3.72 compared with controls with a family history (95% CI, 1.4 to 10) [4]. Patients with simple fibroadenomas and no family history of breast cancer do not have an increased risk. Lobular carcinoma in situ (LCIS) of the breast is commonly identified as an incidental finding in breast biopsies performed because of either a mammographic abnormality or a palpable mass. Lobular neoplasia (LN) includes atypical lobular hyperplasia (ALH) and LCIS. The significance of lobular intraepithelial neoplasia (LIN), which includes lobular carcinoma in situ (LCIS) and ALH of the breast, remains uncertain. LN is often an incidental finding on breast core needle biopsy or open biopsy, and management remains controversial [8]. Initially regarded as a pre-invasive form of breast cancer analogous to ductal carcinoma in situ, LCIS is treated by mastectomy. As evidence mounted for an equal risk of invasive carcinoma in both breasts, bilateral mastectomy was advocated by some authors [9]. More recent studies suggest that LCIS is a marker for increased risk rather than a true precursor of invasive carcinoma, and this allows a more conservative approach [10]. LCIS is associated with an increased risk of breast cancer, ranging from a three- to four-fold increased risk with ALH, and up to an eight- to ten-fold increased risk with LCIS [11,12].

## Case presentation

**Figure 1:**
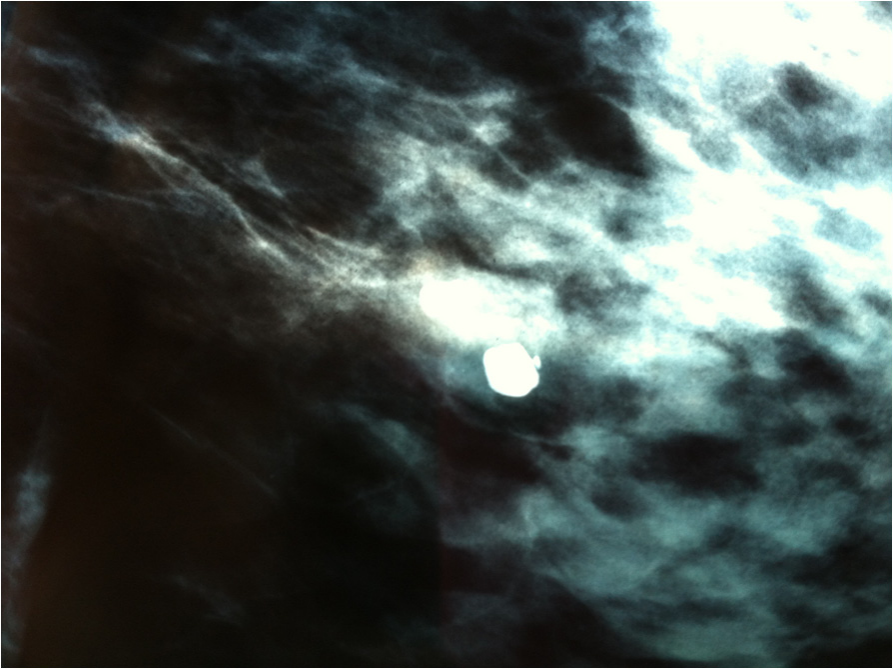
Popcorn-like calcifications shown by mammogram.

**Figure 2:**
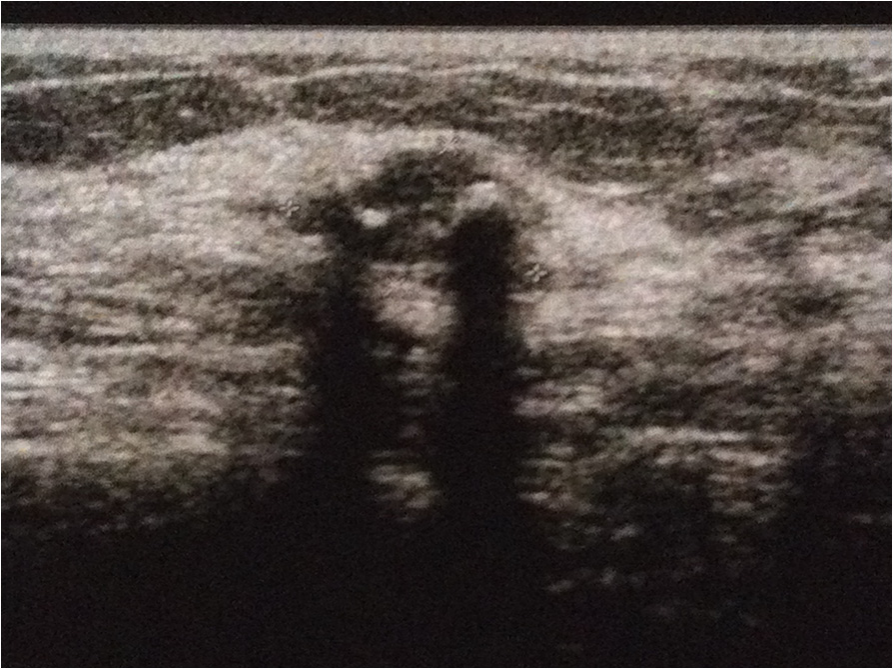
Hypoechoic lump at breast ultrasound.

**Figure 3:**
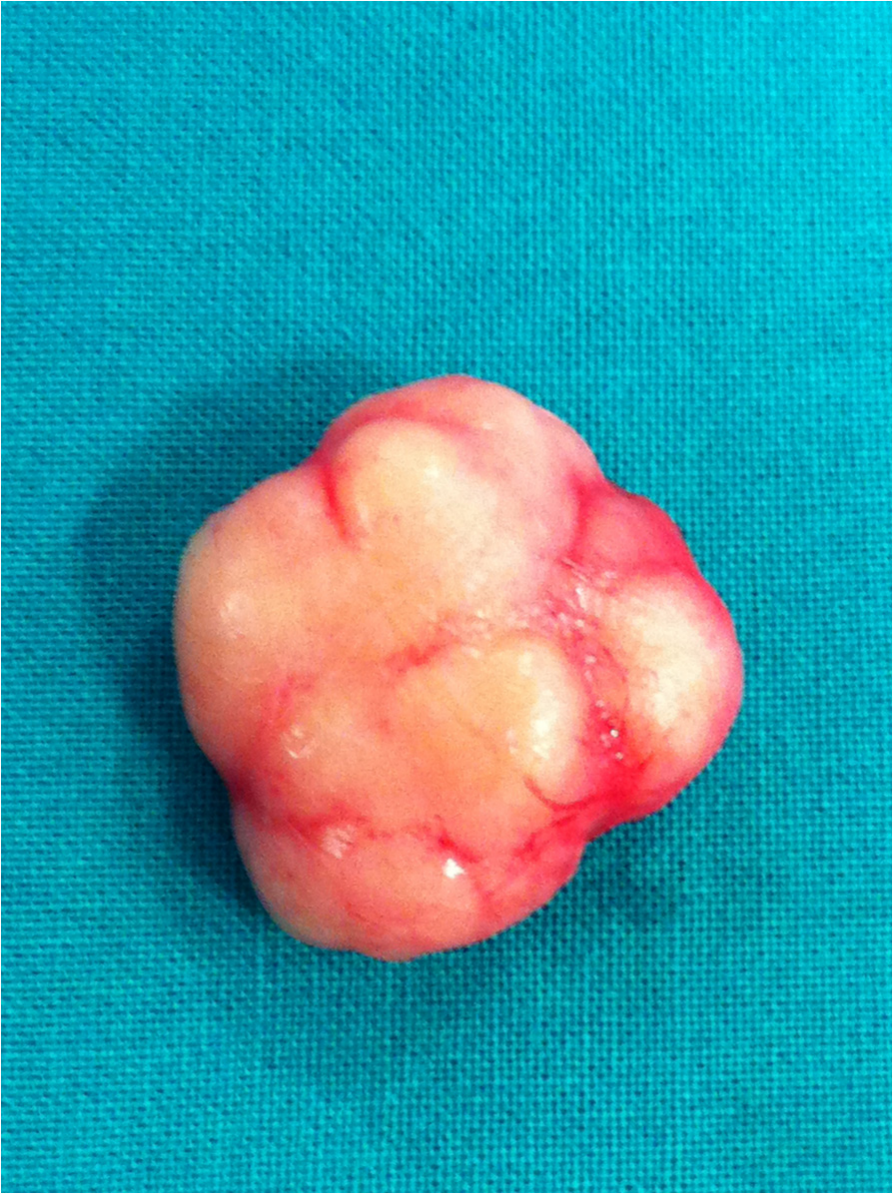
Intraoperative specimen.

**Figure 4:**
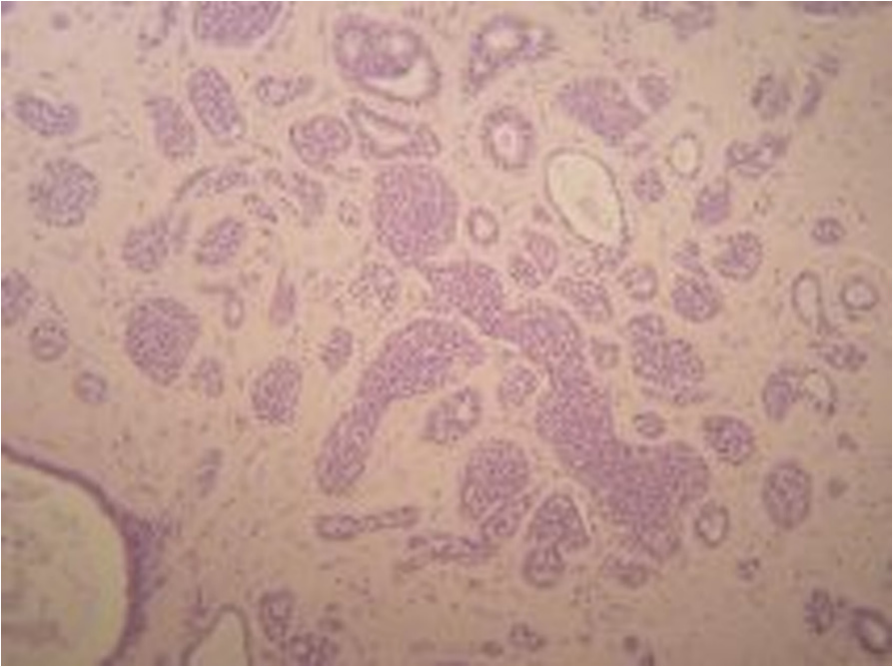
Sample stained with hematoxylin and eosin staining showing a fibroadenoma.

**Figure 5:**
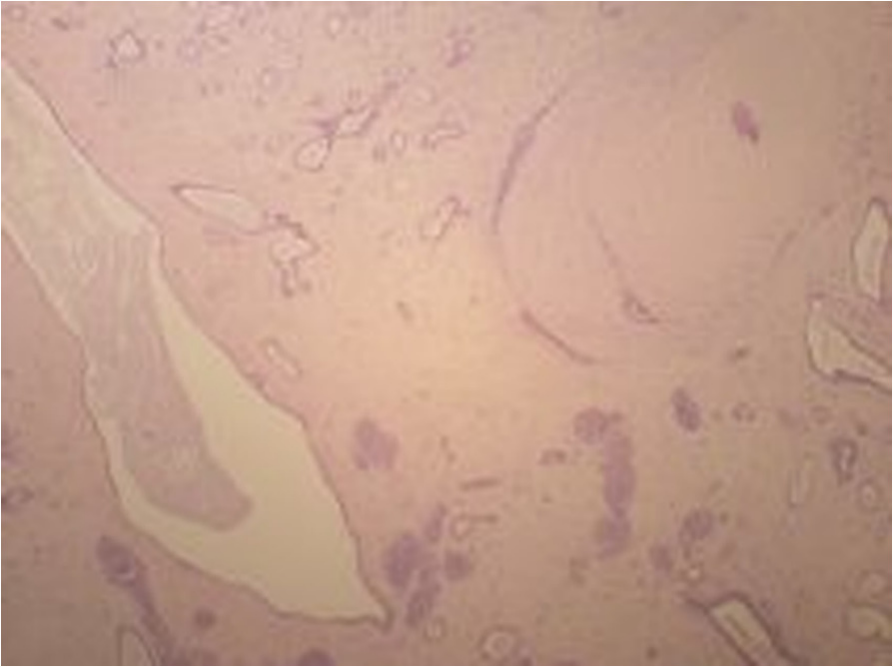
Sample stained with hematoxylin and eosin showing LCIS within the fibroadenoma.

A 44-year-old woman with a familial history of breast cancer presented with a 1.5 cm palpable, smooth, mobile lump in the lower-inner quadrant of her right breast. Standard mediolateral oblique and craniocaudal mammograms showed a cluster of eccentric popcorn-like calcifications within the fibroadenoma (Figure 1). Breast ultrasound showed an oval, smooth bordered hypoechoic lump with some hyperechoic spots (Figure 2). Fine needle aspiration biopsy was negative for malignant cells and showed a complex fibroadenoma cellular pattern. Considering the familial history of cancer, the patient’s age and mammographic calcifications findings, lumpectomy was performed. Intra-operative frozen sections supported our pre-operative clinical-instrumental diagnosis of complex fibroadenoma. The final histological report macroscopically showed a brown-yellowish nodular lesion of 1.5 × 1.3 cm, capsulated with a tough elastic consistency (Figure 3). The fibroadenoma was sliced into 2-mm-thick samples and stained with hematoxylin-eosin. Microscopically, definitive sections confirmed the intra-operative diagnosis of fibroadenoma (Figure 4), but LIN foci were found, surrounded by ALH (Figure 5). LIN constituted an independent entity and was not an extension of the fibroadenoma. Treatment for the patient consisted of only surgery. Six monthly ultrasound, annual mammography, and a clinical follow-up were carried out. After 5 years of followup, neither local recurrence nor contralateral breast cancer ever developed.

## Conclusions

The possibility of an old benign breast lump might be supported by fine needle aspiration biopsy or core biopsy before initiating follow-up. According to our experience, when patients are older than 40 years and have a familial history of breast cancer, we prefer to perform lumpectomy with follow-up to avoid the risk of underestimated in situ foci within the lump. Even if the incidence of carcinoma within fibroadenoma is low, the long term risk of progression toward invasive breast cancer is unknown. The difference between benign and malignant lumps is determined by DNA mutations. The loss of heterozygosity, achieved by the passing of time, appears to be the most important factor to consider during breast intraepithelial neoplasm development. We strongly recommend removing old fibroadenomas, even though they present with “benign-looking calcifications” in patients with a familial history of breast cancer, because of the chance of finding lobular or ductal carcinoma foci within them. LCIS within a breast fibroadenoma is a rare entity. In light of the recent literature showing that carcinomas arising within a fibroadenoma have the same biological behavior as those arising independently, their management should be the same.

## Consent

Written informed consent was obtained from the patient for publication of this case report and any accompanying images. A copy of the written consent is available for review by the Series Editor of this journal.

## Abbreviations

LIN: Lobular intraepithelial neoplasia; ALH: Atypical lobular hyperplasia; LCIS: Lobular carcinoma in situ.

## Competing interests

The authors declare that they have no competing interests.

## Authors’ contributions

GL performed the diagnosis and surgery. EE drafted the manuscript and revised it critically for important intellectual content. VS and RDM gave substantial contributions to conception and design. GC sectioned and stained the specimens. DDR provided mammogram and ultrasound images. PF gave final approval of the version to be published. All authors read and approved the final manuscript.
