# Correction: Lobular intraepithelial neoplasia arising within breast fibroadenoma

**DOI:** 10.1186/1756-0500-7-206

**Published:** 2014-04-01

**Authors:** Gennaro Limite, Emanuela Esposito, Viviana Sollazzo, Giuseppe Ciancia, Cesare Formisano, Rosa Di Micco, Dario De Rosa, Pietro Forestieri

**Affiliations:** 1 University Department of Clinical Medicine and Surgery, Breast Unit, University of Naples Federico II, Naples, Italy; 2 Department of Pathology, University of Naples Federico II, Naples, Italy; 3 Department of Diagnostic Radiology, University of Naples Federico II, Naples, Italy

## 

After publication of our article [1], it has come to our attention that Professor Cesare Formisano was incorrectly excluded from the list of authors in the initial version of this manuscript. We publish this correction to update the author list to include Professor Formisano as an author. The correct author list is as follows:.

Gennaro Limite, Emanuela Esposito, Viviana Sollazzo, Giuseppe Ciancia, Cesare Formisano, Rosa Di Micco, Dario De Rosa and Pietro Forestieri.

We apologise for any inconvenience caused by this error.
